# Perspectives of Autologous Mesenchymal Stem-Cell Transplantation in Macular Hole Surgery: A Review of Current Findings

**DOI:** 10.1155/2019/3162478

**Published:** 2019-02-24

**Authors:** Raffaele Nuzzi, Federico Tridico

**Affiliations:** ^1^S.C.U. Ophthalmology Unit, “City of Health and Science” University Hospital, Via Cherasco 23, 10126 Turin, Italy; ^2^Department of Surgical Sciences, University of Turin, Corso Dogliotti 14, 10126 Turin, Italy

## Abstract

The main treatment available for idiopathic macular holes is represented by pars plana vitrectomy with internal limiting membrane peeling. However, late-stage macular holes are affected by a higher risk of surgical failure. Although adjuvant techniques can be employed, a satisfactory functional recovery is difficult to achieve in refractory macular holes. Given their neuroprotective and antiapoptotic properties, mesenchymal stem cells (MSCs) may represent an appealing approach to treat these extreme cases. The purpose of this review is to highlight the findings regarding healing mechanisms exerted by mesenchymal stem cells and preliminary application in cases of refractory macular holes. When compared with MSCs, MSC-derived exosomes may represent a feasible alternative, given their reduced risk of undesired proliferation and easiness of use.

## 1. Introduction

A macular hole is an anatomic gap in the retina occurring at the level of the fovea. The majority of macular holes is idiopathic, although they can be associated with highly myopic eyes or ocular trauma, especially in the elderly [1].

The general prevalence of full-thickness idiopathic macular holes found in the Beaver Dam Study is 0.3%, with rates increasing to 0.8% in people with 75 or more years of age [2]. The genesis of macular holes is supposed to be promoted by vitreous tractions or due to epiretinal membranes at the level of the fovea.

Occasionally, macular holes are associated with retinal detachments, leading to severe visual impairment, especially in elderly people with elevated myopia and in the presence of a posterior staphyloma [3, 4].

The typical progression of macular holes is characterized by a series of stages (reported by a biomicroscopic analysis by Gass) over a period of weeks, featuring retinal defects which in severe cases involve the macular region in all its thickness [5]. In the later years, optical coherence tomography (OCT) has become a useful method to confirm diagnosis and to better define the staging of macular holes. If left untreated, full-thickness macular holes lead to a poor visual prognosis, with visual acuity of 20/100 in more than 50% of all cases [6–10].

As a treatment for macular holes, Kelly et al. introduced in 1991 the use of pars plana vitrectomy, with removal of epiretinal membranes and introduction of long-acting gas tamponades [11]. Internal membrane peeling has been proposed by Eckardt et al. to further improve anatomical closure rates [12].

Other several advances in this surgical field allowed more satisfactory outcomes with a 98% closure rate of early-onset macular holes [13]. However, treatment of large (>400 *μ*m) and late-diagnosed macular holes (>6 months) are affected by low success rates [14], particularly in highly myopic eyes [15]. In fact, size and stage of macular holes, duration of symptoms, and preoperative visual acuity have been reported as prognostic factors [16]. Moreover, a retrospective chart review by Kim et al. concluded that large basal hole diameter and thin choroid are associated with poorer visual outcomes [17].

As of today, the goal of the surgical procedure focuses on the resolution of vitreal or epiretinal tangential tractions on the central retina and macular hole's margins, achieving its closure with good anatomical results in more than 90% of eyes with full-thickness macular holes [18–20]. Even if a review by Parravano et al. concluded that vitrectomy is effective in improving visual acuity and in achieving hole closure [21], surgical closure rates are relatively low in complex cases, such as large macular holes, myopic macular holes or refractory holes after the first surgery [15, 22–24]. Novel techniques have been introduced to improve anatomical and visual outcomes in complicated cases [25–29]. These techniques include outpatient fluid-gas exchange, the inverted internal limiting membrane (ILM) flap technique, ILM fragment transplantation, and the use of human autologous serum, platelet concentrate, transforming growth factor-beta 2, and heavy silicone oil endotamponades [26, 30–40]. Inverted ILM flap techniques may facilitate the proliferation of glial cells, which then fill the hole and facilitate its closure and are currently employed as a treatment of choice for macular holes in patients with severe myopia and posterior staphylomas. However, even if good anatomical outcomes (with successful macular hole closure) are achieved, functional recovery and regeneration of the retina are difficult to obtain, especially in case of late stages of the disease.

Retinal degenerative diseases can be characterized by loss of cellular elements, such as retinal ganglion cells (RGC), retinal pigment epithelium (RPE) cells, and photoreceptors, and the following visual impairment is typically irreversible, since retinal cells lack self-repair capability. Several cell-based treatments have been proposed and evaluated to overcome these limits.

Cell therapy represents an appealing alternative to obtain regeneration of damaged retina, potentially improving functional outcomes also in surgery of macular hole. Transplantation of retinal cells has been considered as a potential treatment for retinal degenerative diseases, particularly in late stages associated with severe cell damage. This approach aims at replacing lost retinal cells using stem cells, progenitor cells, and mature neural retinal cells with potential applications in a wide range of retinal degenerative conditions.

The purpose of this review is to highlight perspectives of cell-based treatment for refractory macular holes, focusing particularly on the current evidence regarding the application of autologous mesenchymal stem cells (MSCs) since they overcome the ethical and safety problems of embryonic and induced pluripotent stem cells (ESCs and iPSCs, respectively). In addition, mesenchymal stem cells can be easily extracted from different human tissues and possess neuroprotective and immunomodulatory properties.

## 2. Hints of Cell Therapy for Retinal Degenerations

Appealing anatomic and functional outcomes have been reported in animal models of retinal degeneration by various study groups evaluating the transplant of RPE cells, retinal progenitor cells, photoreceptor precursors, or full-thickness retinal sheets [41–44]. However, integration into the receiving retina and functional recovery still represent major obstacles for successful transplant. Submacular transplantation of autologous RPE, retrieved from the peripheral retina, has led to visual acuity improvements in patients affected by macular degeneration [45–48]. However, since ocular autologous cell sources are limited, a treatment based on stem cells seems more feasible, given their potentially unlimited proliferative properties.

In fact, it is possible to obtain a virtually unlimited source of different retinal cells (RPE cells, ganglion cells, and photoreceptors) from the expansion of pluripotent stem cells, both embryonic and iPSCs, as shown in numerous studies [49–53].

Several stem-cell types can differentiate into photoreceptors in preclinical models of human retinal degenerative disease, including bone marrow-derived hematopoietic stem cells, bone marrow-derived MSCs, neural progenitor cells (NPCs), and forebrain-derived progenitor cells [54]. Carr et al. [55] evaluated the injection of human iPSC-derived RPE transplants (iPSC-RPE) in murine models of retinal degeneration, with protective effects on photoreceptors and better optokinetic responses in treated rats when compared with the control group. Finally, photoreceptor rescue effects of RPE derived from iPSC have been observed in retinal degeneration in mice [56].

## 3. Mesenchymal Stem Cells for Retinal Diseases

Mesenchymal stem cells possess the ability of self-renewal and unlimited differentiation. Moreover, it is possible to retrieve MSCs from several human tissues (bone marrow, dental pulp, adipose tissue, etc.). Given their well-known properties and the possibility to isolate MSCs with ease, this cell type represents a promising candidate for the treatment of retinal degenerative diseases [57]. In fact, MSCs feature a good proliferative potential with proven neuroprotective effects and reduced immunogenicity [58]. Furthermore, MSCs and MSC-derived exosomes (MSC-Exos) have been successfully tested for the treatment of retinal inflammation [59–62], injury [63], and degeneration [64, 65].

**Figure 1:**
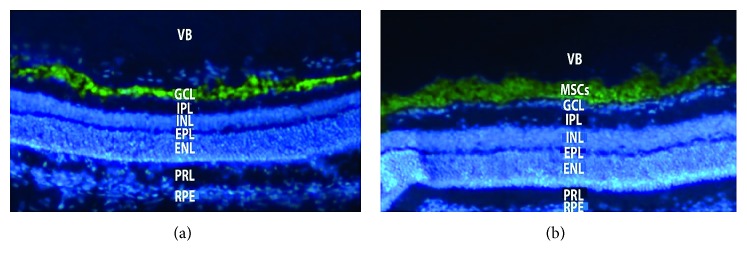
Histological findings revealing integration of mesenchymal stem cells at the level of the ganglion cell layer in the ischemic rat retina. Mesenchymal stem cells were not able to migrate to the ganglion cell layer of nonischemic retina. VB = vitreous body; GCL = ganglion cell layer; IPL = internal plexiform layer; INL = internal nuclear layer; EPL = external plexiform layer; ENL = external nuclear layer; PRL = photoreceptor layer; RPE = retinal pigment epithelium; MSCs = mesenchymal stem cells.

MSCs derived from both bone marrow and adipose tissue have been injected into the subretinal space of animal models of retinal degeneration with significant improvements in terms of visual function and RGC density, although for a short period [66–68]. Other studies indicated that bone marrow-derived MSCs (BMMSCs) might differentiate into photoreceptors cells after their introduction in the subretinal cavity, as observed in rats with hereditary retinal degeneration [69]. Moreover, transplantation of BMMSCs in animal models of retinitis pigmentosa led to preservation of outer nuclear layer cells, with prolonged photoreceptor survival [70]. Our group had performed preliminary evaluations of MSCs intravitreal injections in rat models, highlighting the ability of MSCs to survive, migrate, and integrate at the level of the ganglion cell layer, which improved in case of retinal ischemia (Figure 1) [71].

It is indicated that MSCs can express a variety of factors which could protect the injured retina, such as NGF, CNTF, BDNF, bFGF, NTFs, and IGF1 [67, 72, 73]. The mechanisms which these effects are based on have been extensively described in the literature [74, 75]. Moreover, it has been reported that MSCs secrete also factors modulating perfusion and neovascularization in ischemic retina [76, 77].

The intravitreal use of CD34 + BMMSCs in patients with age-related macular degeneration has been evaluated in three phase I clinical trials, with improved visual acuity over a 12-month follow-up (NCT01518127, NCT01068561, and NCT01518842) [78, 79]. In a phase II clinical trial (NCT01560715), a quality-of-life improvement in patients affected by retinitis pigmentosa was observed, although not sustained at 12 months after MSCs injection [80–82].

However, in a study employing CD34 + BMSCs intravitreal injections (with 3.4 × 106 cells in a 0.1 ml suspension), no improvement in visual function was observed in 6 patients affected by AMD, even if no complications occurred [79, 82]. In a study by Weiss et al., patients with different retinal diseases received varied BMMSCs administration methods (retrobulbar, subtenonian, intravitreal, intraoptic nerve, subretinal, and intravenous) in accordance with different ocular diseases (NCT01920867), with improvements in terms of visual acuity and visual field parameters [83–86].

Severe complications and vision loss, related probably to stem-cell preparation, have been observed in a clinical trial adopting adipose-derived MSCs (NCT02024269), leading to the withdrawal of the study [87]. This discrepancy in outcomes between BMMSCs and adipose-derived MSCs may be due to different quantity and preparation of injectable solution, given the significant differences among the conducted trials.

## 4. Mesenchymal Stem Cells for Macular Holes: Current Evidence

Currently, little evidence is present in the literature regarding the application of mesenchymal stem cells for the treatment of macular holes, in both animal models and humans. In order to assess the feasibility of cell therapy in the treatment of macular holes, Hara et al. and Yamana et al. first applied human adipose-derived MSCs in rabbit models of retinal hole [88, 89], with restoration of anatomic integrity.

Encouraged by these positive findings, Xuqian et al. injected in vitro adipose-derived MSC cultures into rabbits with retinal holes undergoing vitrectomy, evaluating the effects with optical coherence tomography and immunohistochemical analysis. OCT images of treated eyes day showed reattachment of the everted hole edges at the second day after surgery with onset of healing tissue at the 4^th^ postoperative day, in contrast with control eyes in which healing was delayed till the 32^nd^ postoperative day. In addition, final retinal thickness was significantly greater in treated eyes (with values similar to normal retina) in comparison with control eyes (*p*=0.001), with more evident differences at early postoperative time [90].

To confirm the effects of adipose-derived MSCs, additional cell injections were performed in refractory retinal holes after surgery in the control group, with clear observation of a healing process similar to the one occurred in the treatment group.

The histological analysis by Xuqian et al. confirmed that healing tissue cells were different in the retinal holes of the treated group compared to the control group. In fact, control eyes presented a proliferation of collagenous fibrotic tissue, while the retina of treated eyes featured mesenchymal tissue with photoreceptor- and bipolar-like cells. However, it was observed that these cells were not originated from injected MSCs since the transplanted donor cells did not survive abundantly in the healing tissue. Xuqian assumed that the healing process in the transplantation group was promoted by MSCs expression of cytokines with antiapoptotic functions and related to extracellular matrix development, such as intercellular adhesive factors, with better adhesion of the retina to the RPE.

A recent case series of 7 human patients affected by refractory and late-stage macular holes evaluated effects on visual function and safety of MSC suspension and MSC-Exos in combination with pars plana vitrectomy and gas tamponade (SF6 or air in patients receiving exosomes) or heavy silicon oil (in patients receiving cell suspension). Silicon oil was employed in order to limit the dispersion of the cell suspension, and patients were instructed to remain in supine position for 1 day. Silicon oil was then removed with surgery in the two patients receiving the cell suspension [91].

BCVA improvements were observed in five patients presenting hole closure after surgery, with a mean postoperative visual acuity of 20/110 (ranging from 20/160 to 20/50). The complete hole closure was observed at 19 days after surgery, by average (ranging from 3 days in a case receiving MSCs suspension to 30 days in patients receiving intravitreal MSC-Exos). The only patient who did not experience improvements in visual acuity was a woman with a 4-year history of macular hole, which was already treated with previous unsuccessful surgery. Nevertheless, she presented hole closure at 1 month after pars plana vitrectomy with intravitreal MSC-Exos, even if her BCVA remained unchanged. Only one of the treated eyes did not presented a macular hole closure after pars plana vitrectomy in combination with phacoemulsification and intravitreal injection of MSC-Exos, but its BCVA improved slightly (from 20/200 to 20/160) probably due to cataract removal. Among all the patients included in the case series, reduction in central scotoma was observed, with significant improvements in one patient receiving 20 *µ*g of MSC-Exos via intravitreal injection in association with an air tamponade (in accordance with an improvement in BCVA from 20/200 to 20/50).

In regard to adverse reactions, formation of fibrotic epiretinal membrane due to proliferation of injected cells occurred in one patient receiving MSCs in association with pars plana vitrectomy and heavy silicon oil endotamponade. This fibrotic membrane was then removed through an additional surgical procedure, and the pathological analysis revealed the proliferation of fibroblast-like cells. In the following 6 months, no recurrence of fibrotic membrane was observed, and a decrease in central scotoma was reported. Moreover, the first patient who underwent treatment with 50 *μ*g of MSC-Exos presented signs of inflammation in the anterior chamber, which was resolved 3 days after application of steroid eyedrops. Thereafter, MSC-Exos dose was reduced to 20 *μ*g without observation of other cases of significant inflammation. All patients were subjected to a follow-up period lasting between 0.5 and 3 years, without evidence of side effects related to MSCs and MSC-Exos and risk of teratoma development. However, limitations of this preliminary study lie in the small number of patients and the absence of a comparison with a control group, preventing the assessment of the real therapeutic effects of MSCs and MSC-Exos.

## 5. Discussion and Clinical Perspectives

Adjuvant surgical strategies, such as ILM inverted flap and ILM insertion, have been adopted to improve outcomes in refractory and large macular holes with successful anatomical and visual outcomes [42, 92, 93].

However, these techniques are not without potential problems, such the risk of direct damage to RPE. Moreover, the heterogeneous characteristics of late stages and refractory macular holes may prevent the actual assessment of the effects of adjuvant procedures on postoperative results. Among the few studies comparing different adjuvant techniques, the analysis by Park et al. concluded that the inverted flap technique resulted in better recovery of photoreceptor layers with better functional outcomes, when compared with ILM insertion [94].

Stem-cell therapy has emerged as a novel candidate approach for the treatment of retinal degenerative diseases, given their proven effects of neurogenesis, cell replacement, reduced apoptosis, and modulation of inflammation and immune responses [95, 96]. MSCs can be easily retrieved from numerous adult tissues and can potentially differentiate into RPE cells and photoreceptors, providing the possibility of a retinal cell replacement therapy. Moreover, protective effects of MSCs towards RGCs have been demonstrated in an animal model of glaucoma [72].

In case of a macular hole, it is advisable to achieve the contact between the retinal neural epithelium and the RPE, in order to obtain hole closure [89]. Thereafter, adherence of the detached retinal edges to the RPE layer promotes cell migration and proliferation of the overlying retina.

Xuqian et al. in their analysis on animal models concluded that MSCs promote hole closure rate with accelerated healing. Morphological histochemical and OCT observations showed significant differences between repair tissues in eyes receiving MSCs and the reference group, which presented a typical fibrotic scarring [90].

Previous studies have demonstrated that MSCs promote the expression of adhesive factors [97] and extracellular matrix development [98]. In addition, several studies demonstrated the antiapoptotic and protective effects of MSCs, even at the gene level [99, 100]. In addition, MSCs may exert their reparative effects on the retina through material transfer, with improvement in visual function [101, 102].

According to these findings, a possible rationale for future stem-cell therapy may be developed, and failure of macular hole surgery may be explained by long-term detachment of neuroretina from the RPE layer.

In comparison to gene therapy, MSCs provide multiple rescue pathways with a plurality of combined effects. Moreover, in a previous paper published by our group, we observed that the presence of a human retinal pigment epithelial cell-line supernatant helped cells preserve the typical MSC morphology [103]. Therefore, we can hypothesize that RPE can represent an ideal environment in which MSCs can exert their function of cytokines and neurotrophic factors release.

It has been reported that up to 44% of large macular holes remain open after vitrectomy with ILM removal [26] and repeated surgeries in case of failure are associated with a lower closure rate compared with primary surgery [104].

In the only available pilot study, macular hole closure was achieved in six of seven patients who underwent MSC or MSC-Exo therapy, with five patients showing improvements in BCVA. These results suggest that MSCs and MSC-Exos may contribute to hole closure and visual function recovery [91]. MSCs and MSC-Exos can therefore be dedicated to cases of refractory macular holes after first surgery, in which satisfactory results are more difficult to achieve.

Compared with cell suspensions, exosomes are easier to employ, presenting lower immunogenicity and risk of proliferation. It was reported that MSC-Exos have similar protective effects towards the retina after laser-induced lesions and experimental model of uveitis [64, 105].

Since MSC-Exos do not tend to disperse in the vitreal cavity after injection, heavy silicon tamponades and patient positioning may not be relevant to promote their effects. However, the mechanism of MSC-Exo therapy is still not fully understood. It has been suggested that miRNAs carried by MSC-Exos promote neurogenesis and functional recovery [106].

Another issue that must be addressed in larger trials is toxicity of MSCs or MSC-Exo in the treatment of retinal diseases. Among the existing clinical trials, three patients were affected by severe bilateral vision loss after receiving intravitreal injections of autologous adipose tissue derived stem cells, due to severe vitreoretinal proliferation [87] (probably occurred as a result of transformation of injected cells in myofibroblasts-like cells). According to these findings, MSC-Exo therapy may be safer than cell suspension, since cell proliferation is less likely to occur.

Finally, as of today, there is no standardized number and volume for cell injection into the retina. Current clinical trials have injected from 1.68 × 104 to 3.4 × 106 cells in a 100–150 *μ*l suspension, but the exact quantity needed still remains to be discovered.

## 6. Conclusions

The application of mesenchymal stem cells as an adjuvant treatment for refractory and late-stage macular holes can be considered as a promising perspective, in the light of the recent functional and anatomic outcomes in both animal and human trials. Combination of pars plana vitrectomy and MSCs injections or MSCs exosomes application seems feasible, but the evidence regarding their safety needs to be expanded. Nevertheless, autologous mesenchymal stem cells can be extracted from the same patient, which can act both as the donor and the receiving subject, lowering rejection rates and immunological reactions. Furthermore, repeated injections can be performed, if needed. The beneficial effects of MSCs suspensions and exosomes lie in their neuroprotective properties and in the facilitation of macular hole adhesion to the RPE, which promotes healing and closure. These aspects can lead to an effective visual rehabilitation in cases destined to poor results with simple surgery (even if hole closure is achieved). However, MSCs application at the retinal level can lead to unwanted differentiation of transplanted cells with onset of vitreoretinal proliferation, and the presence of preexisting tumors whose growth may be promoted by immunosuppression provided by MSCs represents an important contraindication [107]. Autologous MSCs can be considered safe in terms of immunogenicity and de-novo malignancy development [108]. Finally, long-term clinical trials featuring control groups are still required in order to compare the efficacy of MSCs applications with pars plana vitrectomy and other adjuvant techniques.

## References

[1] Ho A. C., Guyer D. R., Fine S. L. (1998). Macular hole. *Survey of Ophthalmology*.

[2] Klein R., Klein B. E., Wang Q., Moss S. E. (1994). The epidemiology of epiretinal membranes. *Transactions of the American Ophthalmological Society*.

[3] Morita H., Ideta H., Ito K., Yonemoto J., Sasaki K., Tanaka S. (1991). Causative factors of retinal detachment in macular holes. *Retina*.

[4] Akiba J., Konno S., Yoshida A. (1999). Retinal detachment associated with a macular hole in severely myopic eyes. *American Journal of Ophthalmology*.

[5] Gass J. D. M. (1995). Reappraisal of biomicroscopic classification of stages of development of a macular hole. *American Journal of Ophthalmology*.

[6] Casuso L. A., Scott I. U., Flynn H. W. (2001). Long-term follow-up of unoperated macular holes. *Ophthalmology*.

[7] Chew E. Y., Sperduto R. D., Hiller R. (1999). Clinical course of macular holes. *Archives of Ophthalmology*.

[8] Hikichi T., Trempe C. L. (1993). Risk of decreased visual acuity in full-thickness idiopathic macular holes. *American Journal of Ophthalmology*.

[9] Lewis M. L., Cohen S. M., Smiddy W. E., Gass J. D. M. (1996). Bilaterality of idiopathic macular holes. *Graefe’s Archive for Clinical and Experimental Ophthalmology*.

[10] Morgan C. M., Schatz H. (1985). Idiopathic macular holes. *American Journal of Ophthalmology*.

[11] Kelly N. E., Wendel R. T. (1991). Vitreous surgery for idiopathic macular holes. *Archives of Ophthalmology*.

[12] Eckardt C., Eckardt U., Groos S., Luciano L., Reale E. (1997). Entfernung der Membrana limitans interna bei Makulalöchern. *Der Ophthalmologe*.

[13] Michalewska Z., Michalewski J., Cisiecki S., Adelman R., Nawrocki J. (2008). Correlation between foveal structure and visual outcome following macular hole surgery: a spectral optical coherence tomography study. *Graefeʼs Archive for Clinical and Experimental Ophthalmology*.

[14] Ezra E., Gregor Z. J. (2004). Surgery for idiopathic full-thickness macular hole. *Archives of Ophthalmology*.

[15] Suda K., Hangai M., Yoshimura N. (2011). Axial length and outcomes of macular hole surgery assessed by spectral-domain optical coherence tomography. *American Journal of Ophthalmology*.

[16] Ullrich S., Haritoglou C., Gass C. (2002). Macular hole size as a prognostic factor in macular hole surgery. *British Journal of Ophthalmology*.

[17] Kim S. H., Kim H. K., Yang J. Y., Lee S. C., Kim S. S. (2018). Visual recovery after macular hole surgery and related prognostic factors. *Korean Journal of Ophthalmology*.

[18] Margherio A., Margherio R. R., Hartzer M., Trese M. T., Williams G. A., Ferrone P. J. (1998). Plasmin enzyme-assisted vitrectomy in traumatic pediatric macular holes. *Ophthalmology*.

[19] Polk T. D., Smiddy W. E., Flynn H. W. (1996). Bilateral visual function after macular hole surgery. *Ophthalmology*.

[20] Smiddy W. E., Pimentel S., Williams G. A. (1997). Macular hole surgery without using adjunctive additives. *Ophthalmic Surgery and Lasers*.

[21] Parravano M., Giansanti F., Eandi C. M., Yap Y. C., Rizzo S., Virgili G. (2015). Vitrectomy for idiopathic macular hole. *Cochrane Database of Systematic Reviews*.

[22] Stec L. A., Ross R. D., Williams G. A., Trese M. T., Margherio R. R., Cox M. S. (2004). Vitrectomy for chronic macular holes. *Retina*.

[23] Salter A. B., Folgar F. A., Weissbrot J., Wald K. J. (2012). Macular hole surgery prognostic success rates based on macular hole size. *Ophthalmic Surgery, Lasers, and Imaging*.

[24] Scott R. A. H., Ezra E., West J. F. (2000). Visual and anatomical results of surgery for long standing macular holes. *British Journal of Ophthalmology*.

[25] Kuriyama S., Hayashi H., Jingami Y., Kuramoto N., Akita J., Matsumoto M. (2013). Efficacy of inverted internal limiting membrane flap technique for the treatment of macular hole in high myopia. *American Journal of Ophthalmology*.

[26] Michalewska Z., Michalewski J., Adelman R. A., Nawrocki J. (2010). Inverted internal limiting membrane flap technique for large macular holes. *Ophthalmology*.

[27] Michalewska Z., Michalewski J., Dulczewska-Cichecka K., Adelman R. A., Nawrocki J. (2015). Temporal inverted internal limiting membrane flap technique versus classic inverted internal limiting membrane flap technique. *Retina*.

[28] Michalewska Z., Michalewski J., Dulczewska-Cichecka K., Nawrocki J. (2014). Inverted internal limiting membrane flap technique for surgical repair of myopic macular holes. *Retina*.

[29] Shin M. K., Park K. H., Park S. W., Byon I. S., Lee J. E. (2014). Perfluoro-n-octane-assisted single-layered inverted internal limiting membrane flap technique for macular hole surgery. *Retina*.

[30] Del Priore L. V., Kaplan H. J., Bonham R. D. (1994). Laser photocoagulation and fluid-gas exchange for recurrent macular hole. *Retina*.

[31] Johnson R. N., McDonald H. R., Schatz H., Ai E. (1997). Outpatient postoperative fluid-gas exchange after early failed vitrectomy surgery for macular hole. *Ophthalmology*.

[32] Ohana E., Blumenkranz M. S. (1998). Treatment of reopened macular hole after vitrectomy by laser and outpatient fluid-gas exchange11Neither author has any commercial interest in the methods or products described in this manuscript. *Ophthalmology*.

[33] Liggett P. E., Skolik D. S. A., Horio B., Saito Y., Alfaro V., Mieler W. (1995). Human autologous serum for the treatment of full-thickness macular holes. *Ophthalmology*.

[34] Wells J. A., Gregor Z. J. (1996). Surgical treatment of full-thickness macular holes using autologous serum. *Eye (Lond)*.

[35] Kim J. Y., Kwon O. W. (2015). Vitrectomy for refractory macular hole. *Retinal Cases and Brief Reports*.

[36] Kozy D. W., Maberley A. L. (1996). Closure of persistent macular holes with human recombinant transforming growth factor-beta 2. *Canadian Journal of Ophthalmology*.

[37] Rizzo S., Belting C., Genovesi-Ebert F., Cresti F., Vento A., Martini R. (2006). Successful treatment of persistent macular holes using “heavy silicone oil” as intraocular tamponade. *Retina*.

[38] Cillino S., Cillino G., Ferraro L. L., Casuccio A. (2016). Treatment of persistently open macular holes with heavy silicone oil (Densiron 68) versus C2f6. A prospective randomized study. *Retina*.

[39] Morizane Y., Shiraga F., Kimura S. (2014). Autologous transplantation of the internal limiting membrane for refractory macular holes. *American Journal of Ophthalmology*.

[40] De Novelli F. J., Preti R. C., Ribeiro Monteiro M. L., Pelayes D. E., Junqueira Nóbrega M., Takahashi W. Y. (2015). Autologous internal limiting membrane fragment transplantation for large, chronic, and refractory macular holes. *Ophthalmic Research*.

[41] Alexander P., Thomson H. A. J., Luff A. J., Lotery A. J. (2015). Retinal pigment epithelium transplantation: concepts, challenges and future prospects. *Eye*.

[42] Nazari H., Zhang L., Zhu D. (2015). Stem cell based therapies for age-related macular degeneration: the promises and the challenges. *Progress in Retinal and Eye Research*.

[43] Jayakody S. A., Gonzalez-Cordero A., Ali R. R., Pearson R. A. (2015). Cellular strategies for retinal repair by photoreceptor replacement. *Progress in Retinal and Eye Research*.

[44] Seiler M. J., Aramant R. B. (2012). Cell replacement and visual restoration by retinal sheet transplants. *Progress in Retinal and Eye Research*.

[45] Binder S., Krebs I., Hilgers R.-D. (2004). Outcome of transplantation of autologous retinal pigment epithelium in age-related macular degeneration: a prospective trial. *Investigative Opthalmology & Visual Science*.

[46] Binder S., Stanzel B. V., Krebs I., Glittenberg C. (2007). Transplantation of the RPE in AMD. *Progress in Retinal and Eye Research*.

[47] Binder S., Stolba U., Krebs I. (2002). Transplantation of autologous retinal pigment epithelium in eyes with foveal neovascularization resulting from age-related macular degeneration: a pilot study. *American Journal of Ophthalmology*.

[48] van Meurs J. C., Ter Averst E., Hofland L. J. (2004). Autologous peripheral retinal pigment epithelium translocation in patients with subfoveal neovascular membranes. *British Journal of Ophthalmology*.

[49] Idelson M., Alper R., Obolensky A. (2009). Directed differentiation of human embryonic stem cells into functional retinal pigment epithelium cells. *Cell Stem Cell*.

[50] Jin Z.-B., Okamoto S., Mandai M., Takahashi M. (2009). Induced pluripotent stem cells for retinal degenerative diseases: a new perspective on the challenges. *Journal of Genetics*.

[51] Jin Z. B., Takahashi M., Dunnett S. B., Bjorklund A. (2012). Generation of retinal cells from pluripotent stem cells. *Functional Neural Transplantation III Primary and Stem Cell Therapies for Brain Repair, Pt Ii*.

[52] Klimanskaya I., Hipp J., Rezai K. A., West M., Atala A., Lanza R. (2004). Derivation and comparative assessment of retinal pigment epithelium from human embryonic stem cells using transcriptomics. *Cloning and Stem Cells*.

[53] Lund R. D., Wang S., Klimanskaya I. (2006). Human embryonic stem cell-derived cells rescue visual function in dystrophic RCS rats. *Cloning and Stem Cells*.

[54] Zarbin M. (2016). Cell-based therapy for degenerative retinal disease. *Trends in Molecular Medicine*.

[55] Carr A.-J., Vugler A. A., Hikita S. T. (2009). Protective effects of human iPS-derived retinal pigment epithelium cell transplantation in the retinal dystrophic rat. *PLoS One*.

[56] Sun J., Mandai M., Kamao H. (2015). Protective effects of human iPS-derived retinal pigmented epithelial cells in comparison with human mesenchymal stromal cells and human neural stem cells on the degenerating retina inrd1mice. *Stem Cells*.

[57] Jin Z.-B., Gao M.-L., Deng W.-L. (2018). Stemming retinal regeneration with pluripotent stem cells. *Progress in Retinal and Eye Research*.

[58] Ding D.-C., Shyu W.-C., Lin S.-Z. (2011). Mesenchymal stem cells. *Cell Transplantation*.

[59] Zhang L., Zheng H., Shao H. (2014). Long-term therapeutic effects of mesenchymal stem cells compared to dexamethasone on recurrent experimental autoimmune uveitis of rats. *Investigative Opthalmology & Visual Science*.

[60] Chen X., Shao H., Zhi Y. (2016). CD73 pathway contributes to the immunosuppressive ability of mesenchymal stem cells in intraocular autoimmune responses. *Stem Cells and Development*.

[61] Li G., Yuan L., Ren X. (2013). The effect of mesenchymal stem cells on dynamic changes of T cell subsets in experimental autoimmune uveoretinitis. *Clinical and Experimental Immunology*.

[62] Zhang X., Ren X., Li G. (2011). Mesenchymal stem cells ameliorate experimental autoimmune uveoretinitis by comprehensive modulation of systemic autoimmunity. *Investigative Opthalmology & Visual Science*.

[63] Tassoni A., Gutteridge A., Barber A. C., Osborne A., Martin K. R. (2015). Molecular mechanisms mediating retinal reactive gliosis following bone marrow mesenchymal stem cell transplantation. *Stem Cells*.

[64] Yu B., Shao H., Su C. (2016). Exosomes derived from MSCs ameliorate retinal laser injury partially by inhibition of MCP-1. *Scientific Reports*.

[65] Machalinska A., Kawa M., Pius-Sadowska E. (2013). Long-term neuroprotective effects of NT-4-engineered mesenchymal stem cells injected intravitreally in a mouse model of acute retinal injury. *Investigative Opthalmology & Visual Science*.

[66] Emre E., Yüksel N., Duruksu G. (2015). Neuroprotective effects of intravitreally transplanted adipose tissue and bone marrow-derived mesenchymal stem cells in an experimental ocular hypertension model. *Cytotherapy*.

[67] Johnson T. V., Bull N. D., Hunt D. P., Marina N., Tomarev S. I., Martin K. R. (2010). Neuroprotective effects of intravitreal mesenchymal stem cell transplantation in experimental glaucoma. *Investigative Opthalmology & Visual Science*.

[68] Harper M. M., Grozdanic S. D., Blits B. (2011). Transplantation of BDNF-secreting mesenchymal stem cells provides neuroprotection in chronically hypertensive rat eyes. *Investigative Opthalmology & Visual Science*.

[69] Kicic A., Shen W.-Y., Wilson A. S., Constable I. J., Robertson T., Rakoczy P. E. (2003). Differentiation of marrow stromal cells into photoreceptors in the rat eye. *The Journal of Neuroscience*.

[70] Arnhold S., Absenger Y., Klein H., Addicks K., Schraermeyer U. (2007). Transplantation of bone marrow-derived mesenchymal stem cells rescue photoreceptor cells in the dystrophic retina of the rhodopsin knockout mouse. *Graefe’s Archive for Clinical and Experimental Ophthalmology*.

[71] Nuzzi R. (2010). *Prospettive Di Terapia Cellulare Oculare. Communication Presented at the XI Meeting of Low Vision Academy*.

[72] Hu Y., Tan H. B., Wang X. M., Rong H., Cui H. P., Cui H. (2013). Bone marrow mesenchymal stem cells protect against retinal ganglion cell loss in aged rats with glaucoma. *Clinical Interventions in Aging*.

[73] Torrente Y., Polli E. (2008). Mesenchymal stem cell transplantation for neurodegenerative diseases. *Cell Transplantation*.

[74] Cselenyak A., Pankotai E., Horváth E. M., Kiss L., Lacza Z. (2010). Mesenchymal stem cells rescue cardiomyoblasts from cell death in an in vitro ischemia model via direct cell-to-cell connections. *BMC Cell Biology*.

[75] Mead B., Logan A., Berry M., Leadbeater W., Scheven B. A. (2017). Concise review: dental pulp stem cells: a novel cell therapy for retinal and central nervous system repair. *Stem Cells*.

[76] Kinnaird T., Stabile E., Burnett M. S. (2004). Local delivery of marrow-derived stromal cells augments collateral perfusion through paracrine mechanisms. *Circulation*.

[77] Tang J., Xie Q., Pan G., Wang J., Wang M. (2006). Mesenchymal stem cells participate in angiogenesis and improve heart function in rat model of myocardial ischemia with reperfusion. *European Journal of Cardio-Thoracic Surgery*.

[78] Siqueira R. C., Messias A., Gurgel V. P., Simões B. P., Scott I. U., Jorge R. (2015). Improvement of ischaemic macular oedema after intravitreal injection of autologous bone marrow-derived haematopoietic stem cells. *Acta Ophthalmologica*.

[79] Cotrim C., Toscano L., Messias A., Jorge R., Siqueira R. (2017). Intravitreal use of bone marrow mononuclear fraction containing CD34+ stem cells in patients with atrophic age-related macular degeneration. *Clinical Ophthalmology*.

[80] Siqueira R. C., Messias A., Messias K. (2015). Quality of life in patients with retinitis pigmentosa submitted to intravitreal use of bone marrow-derived stem cells (Reticell -clinical trial). *Stem Cell Research & Therapy*.

[81] Siqueira R. C., Messias A., Voltarelli J. C., Messias K., Arcieri R. S., Jorge R. (2013). Resolution of macular oedema associated with retinitis pigmentosa after intravitreal use of autologous BM-derived hematopoietic stem cell transplantation. *Bone Marrow Transplantation*.

[82] Park S. S., Bauer G., Abedi M. (2015). Intravitreal autologous bone marrow CD34+ cell therapy for ischemic and degenerative retinal disorders: preliminary phase 1 clinical trial findings. *Investigative Ophthalmology & Visual Science*.

[83] Weiss J., Benes S., Levy S. (2016). Stem cell ophthalmology treatment study (SCOTS): improvement in serpiginous choroidopathy following autologous bone marrow derived stem cell treatment. *Neural Regeneration Research*.

[84] Weiss J. N., Levy S., Benes S. C. (2015). Stem Cell Ophthalmology Treatment Study (SCOTS) for retinal and optic nerve diseases: a case report of improvement in relapsing auto-immune optic neuropathy. *Neural Regeneration Research*.

[85] Weiss J. N., Levy S., Benes S. C. (2016). Stem cell ophthalmology treatment study (SCOTS): bone marrow-derived stem cells in the treatment of Leberʼs hereditary optic neuropathy. *Neural Regeneration Research*.

[86] Weiss J. N., Levy S., Malkin A. (2015). Stem Cell Ophthalmology Treatment Study (SCOTS) for retinal and optic nerve diseases: a preliminary report. *Neural Regeneration Research*.

[87] Kuriyan A. E., Albini T. A., Townsend J. H. (2017). Vision loss after intravitreal injection of autologous “stem cells” for AMD. *New England Journal of Medicine*.

[88] Hara S., Sakuraba T., Nakazawa M. (2000). Morphological changes of retinal pigment epithelial and glial cells at the site of experimental retinal holes. *Graefe’s Archive for Clinical and Experimental Ophthalmology*.

[89] Yamana T., Kita M., Ozaki S., Negi A., Honda Y. (2000). The process of closure of experimental retinal holes in rabbit eyes. *Graefe’s Archive for Clinical and Experimental Ophthalmology*.

[90] Xuqian W., Kanghua L., WeiHong Y. (2011). Intraocular transplantation of human adipose-derived mesenchymal stem cells in a rabbit model of experimental retinal holes. *Ophthalmic Research*.

[91] Zhang X., Liu J., Yu B., Ma F., Ren X., Li X. (2018). Effects of mesenchymal stem cells and their exosomes on the healing of large and refractory macular holes. *Graefe’s Archive for Clinical and Experimental Ophthalmology*.

[92] Pires J., Nadal J., Gomes N. L. (2017). Internal limiting membrane translocation for refractory macular holes. *British Journal of Ophthalmology*.

[93] Lee S. M., Kwon H. J., Park S. W. (2017). Microstructural changes in the fovea following autologous internal limiting membrane transplantation surgery for large macular holes. *Acta Ophthalmologica*.

[94] Park J. H., Lee S. M., Park S. W., Lee J. E., Byon I. S. (2018). Comparative analysis of large macular hole surgery using an internal limiting membrane insertion versus inverted flap technique. *British Journal of Ophthalmology*.

[95] Maltman D. J., Hardy S. A., Przyborski S. A. (2011). Role of mesenchymal stem cells in neurogenesis and nervous system repair. *Neurochemistry International*.

[96] Joyce N., Annett G., Wirthlin L., Olson S., Bauer G., Nolta J. A. (2010). Mesenchymal stem cells for the treatment of neurodegenerative disease. *Regenerative Medicine*.

[97] De Ugarte D. A., Alfonso Z., Zuk P. A. (2003). Differential expression of stem cell mobilization-associated molecules on multi-lineage cells from adipose tissue and bone marrow. *Immunology Letters*.

[98] Roebuck K. A., Finnegan A. (1999). Regulation of intercellular adhesion molecule-1 (CD54) gene expression. *Journal of Leukocyte Biology*.

[99] Rehman J., Traktuev D., Li J. (2004). Secretion of angiogenic and antiapoptotic factors by human adipose stromal cells. *Circulation*.

[100] Otani A., Dorrell M. I., Kinder K. (2004). Rescue of retinal degeneration by intravitreally injected adult bone marrow-derived lineage-negative hematopoietic stem cells. *Journal of Clinical Investigation*.

[101] Pearson R. A., Gonzalez-Cordero A., West E. L. (2016). Donor and host photoreceptors engage in material transfer following transplantation of post-mitotic photoreceptor precursors. *Nature Communications*.

[102] Yeo R. W. Y., Lai R. C., Zhang B. (2013). Mesenchymal stem cell: an efficient mass producer of exosomes for drug delivery. *Advanced Drug Delivery Reviews*.

[103] Nuzzi R., Gunetti M., Rustichelli D. (2012). Effect of in vitro exposure of corticosteroid drugs, conventionally used in AMD treatment, on mesenchymal stem cells. *Stem Cells International*.

[104] Hillenkamp J., Kraus J., Framme C. (2007). Retreatment of full-thickness macular hole: predictive value of optical coherence tomography. *British Journal of Ophthalmology*.

[105] Bai L., Shao H., Wang H. (2017). Effects of mesenchymal stem cell-derived exosomes on experimental autoimmune uveitis. *Scientific Reports*.

[106] Xin H., Li Y., Chopp M. (2014). Exosomes/miRNAs as mediating cell-based therapy of stroke. *Frontiers in Cellular Neuroscience*.

[107] Volarevic V., Markovic B. S., Gazdic M. (2018). Ethical and safety issues of stem cell-based therapy. *International Journal of Medical Sciences*.

[108] Lalu M. M., McIntyre L., Pugliese C. (2012). Safety of cell therapy with mesenchymal stromal cells (SafeCell): a systematic review and meta-analysis of clinical trials. *PLoS One*.

